# A Novel Prognostic Model for Patients with Primary Gastric Diffuse Large B-Cell Lymphoma

**DOI:** 10.1155/2022/9636790

**Published:** 2022-10-27

**Authors:** Guangrong Lu, Zijian Lin, Yejiao Ruan, He Huang, Jiafeng Lin, Jialin Pan

**Affiliations:** ^1^Department of Gastroenterology, The Second Affiliated Hospital and Yuying Children's Hospital of Wenzhou Medical University, Wenzhou, Zhejiang, China; ^2^Department of Hematology, The Second Affiliated Hospital and Yuying Children's Hospital of Wenzhou Medical University, Wenzhou, Zhejiang, China; ^3^Department of Cardiology, The Second Affiliated Hospital and Yuying Children's Hospital of Wenzhou Medical University, Wenzhou, Zhejiang, China

## Abstract

**Objectives:**

Primary gastric diffuse large B-cell lymphoma (PG-DLBCL) is a common phenotype of extranodal non-Hodgkin's lymphoma (NHL). This research aims to identify a model for predicting overall survival (OS) and cancer-specific survival (CSS) in PG-DLBCL.

**Methods:**

A total of 1716 patients diagnosed with PG-DLBCL between 1975 and 2017 were obtained from the SEER database and further randomly divided into the training and validating cohorts at a ratio of 7 : 3. Univariate and multivariate cox analyses were conducted to determine significant variables for the construction of nomogram. The performance of the model was then assessed by the concordance index (C-index), the calibration plot, and the area under the receiver operating characteristic (ROC) curve (AUC).

**Results:**

Multivariate analysis revealed that age, race, insurance status, Ann Arbor stage, marital status, chemotherapy, and radiation therapy all showed a significant association with OS and CSS. These characteristics were applied to build a nomogram. In the training cohort, the discrimination of nomogram for OS and CSS prediction was excellent (C-index = 0.764, 95% CI, 0.744–0.784 and C-index = 0.756, 95% CI, 0.732–0.780). The AUC of the nomogram for predicting 3- and 5-year OS was 0.779 and 0.784 and CSS was 0.765 and 0.772. Similar results were also observed in the internal validation set.

**Conclusions:**

We have successfully established a novel nomogram for predicting OS and CSS in PG-DLBCL patients with good accuracy, which can help physicians to quickly and accurately complete the evaluation of survival probability, risk stratification, and therapeutic strategy at diagnosis.

## 1. Introduction 

The stomach is the most commonly involved organ in extranodal non-Hodgkin lymphoma (NHL), and diffuse large B-cell lymphoma (DLBCL) is the most common histological type with a prevalence estimated at 40–70% [1–3]. The International Prognostic Index (IPI) and Lugano stage systems are widely used tools to stage gastrointestinal lymphomas to plan for therapy and surveillance. However, primary gastric DLBCL (PG-DLBCL) is usually diagnosed with low or intermediate IPI, and the prognosis is not consistent with patients with nodal or other extranodal lesions [4]. And since rituximab was approved by the FDA in 1997, the outcomes of DLBCL were significantly improved. Therefore, as the present predictive scoring systems are limited, the detailed information obtained for each patient, including age at diagnosis, sex, race, marriage, Ann Arbor stage, primary site, surgery, molecular subclassification, genetic abnormality, and insurance status were recommended to be collected and analyzed to perform a novel predictive nomogram.

Based on the Surveillance, Epidemiology, and End Results (SEER) database of the National Cancer Institute, population-level multiparameters or factors were analyzed and developed to be a predictive nomogram for prognosis among patients with newly diagnosed lymphoma. SEER is one of the most representative large tumor databases in North America, which provides a broad path for the study of malignant tumors and rare tumors. These prognostic risk parameters are explored from SEER database following a series process. And the prognostic nomogram comprising those parameters always performs more accurate comparing to the traditional or current survival-analysis tools [5, 6]. Here, we integrated various types of prognostic parameters, including the well-established demographic and baseline clinical characteristics, primary sites, race, surgery, and chemotherapy, to develop and establish a new model predicting the overall survival (OS) and cancer-specific survival (CSS) of PG-DLBCL patients.

## 2. Methods

### 2.1. Data Source

**Figure 1:**
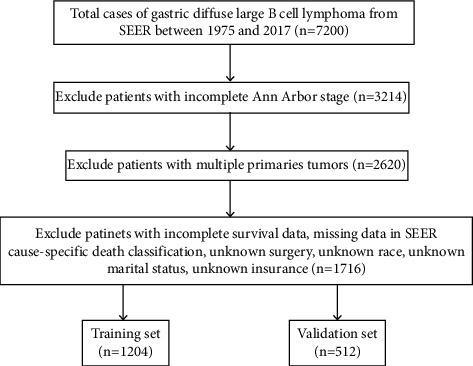
Flow diagram of the PG-DLBCL patients with training and validation cohorts.

The data of PG-DLBCL patients (between 1975 and 2017) were screened from the SEER registry database of the National Cancer Institute using SEER*∗*Stat software (version. 8.3.5). As all of the data in this study were obtained from the SEER database with a publicly available method, no local ethical approval or declaration was required for this study. The information of total 7200 patients were collected following SEER variables: age at diagnosis, sex, race, marital status, insurance type, Ann Arbor stage, surgery, chemotherapy, radiation therapy, and survival time. The exclusion criteria include the following: (1) cases with incomplete Ann Arbor staging at diagnosis or with other multiple primary tumors, (2) missing or incomplete information of follow-up, (3) unclear characteristic data above. A total of 1716 gastric-DLBCL patients were randomly divided into training set and validation set at a ratio of 7 : 3, which are 1204 and 512 cases, respectively (Figure 1).

### 2.2. Construction and Validation of the Nomogram

We incorporated the characteristics of the training cohort to establish the nomogram. The endpoints were OS and CSS, which were measured from the date of first diagnosis to the date of any cause of death. Survival was estimated using the Kaplan–Meier method and Cox regression analysis. Univariate and multivariate analyses were performed to determine independent prognostic variables, and the factors observed to have significant associations with OS or CSS were applied to construct the nomogram. Next, internal validation was performed. The performance of the nomogram was measured by Harrell's concordance index (C-index) and the area receiver operating characteristic (ROC) curve (AUC) [7]. Finally, comparisons between the nomogram and the Ann Arbor stage system were evaluated by C-index and AUC.

### 2.3. Statistical Analysis

All the data were analyzed using R version 3.4.2 software (the R Foundation for Statistical Computing, Vienna, Austria. http://www.r-project.org). The bilateral *P* < 0.05 was regarded as significant.

## 3. Results

### 3.1. Clinical Characteristics

Clinical characteristics of the training and validation cohorts were shown in Table 1. In the training cohort, the majority of patients were over 60 years old (70.0%), male (58.1%), White (78.5%), married (56%), and insured (78.7%). Furthermore, Ann Arbor stages I, II, III, and IV accounted for 40.9, 23.5, 8.9, and 26.7% of all the cases, respectively. Most patients (75.8%) experienced chemotherapy, while just 9.7% and 15.7% patients received surgery and radiation therapy, respectively. Overall, patients in the two sets shared similar clinical characteristics (*P* > 0.05).

### 3.2. Prognostic Factors in the Training Cohort

The results of the univariate and multivariate analysis are listed in Table 2. In the two multivariate analyses, age, race, marital status, insurance status, Ann Arbor stage, chemotherapy, and radiation therapy were significantly associated with OS. However, surgery treatment was evaluated as a nonsignificant factor with *P* value >0.05. In addition, we analyzed the association of each parameter with the CSS of patients in the training cohort, and found significant prognostic factors consistent with the OS generally.

### 3.3. Construction of Nomogram

**Figure 2:**
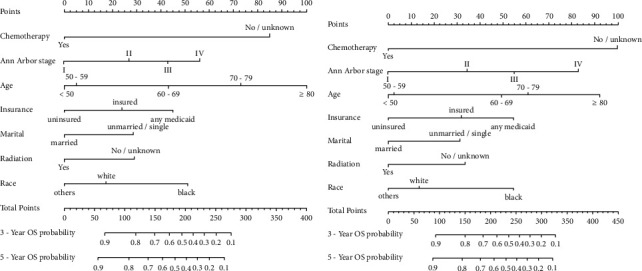
Nomograms predicting 3- and 5-year OS (a) and CSS (b) of patients with PG-DLBCL.

The prognostic nomogram for 3- and 5-year OS is shown in Figure 2. By adding up the scores for each selected variable, a patient's probability of individual survival can be easily calculated. The OS was better for younger patients (particular the patients under 60 years old), patients with early Ann Arbor stage, uninsured and married patients. Furthermore, the patients who had chemotherapy or radiation therapy also exhibited better OS probability. Here, we found Black patients performed the worst OS compared to White and other ethnic patients. In addition, the prognostic nomogram for 3- and 5-year CSS of gastric-DLBCL patients was similar to OS in general.

### 3.4. Validation of Nomogram

**Figure 3:**
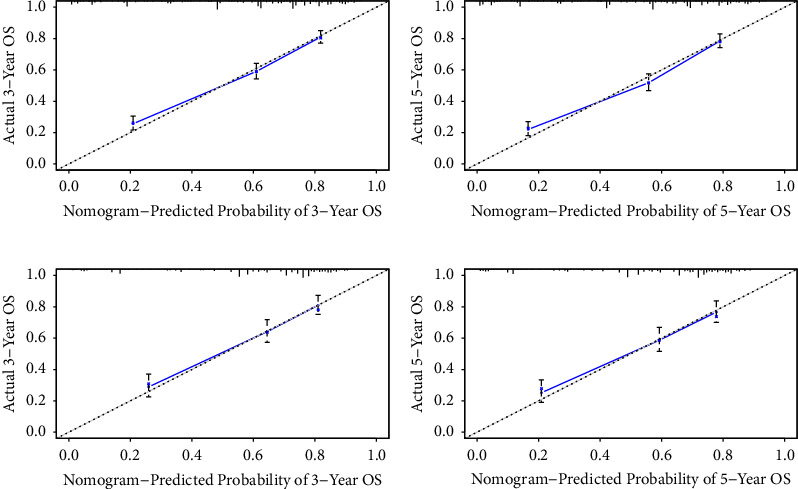
Calibration plots of the nomogram for 3- and 5-year OS prediction of the training cohort (a, b) and internal validation cohort (c, d).

**Figure 4:**
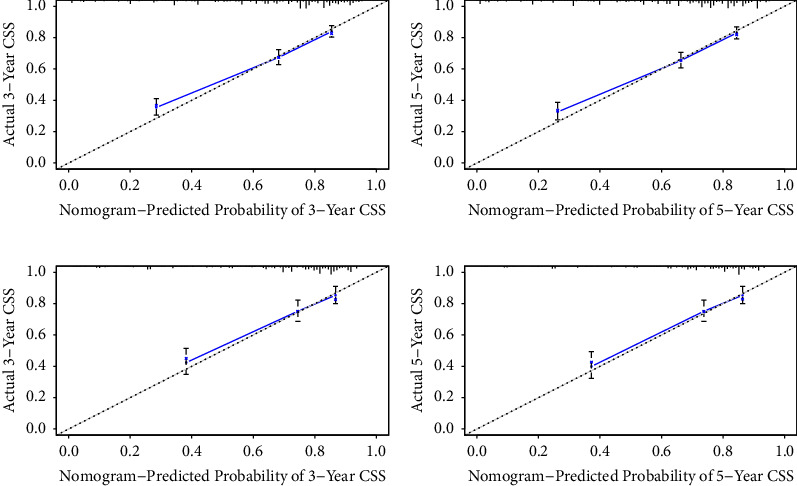
Calibration plots of the nomogram for 3- and 5-year CSS prediction of the training cohort (a, b) and internal validation cohort (c, d).

**Figure 5:**
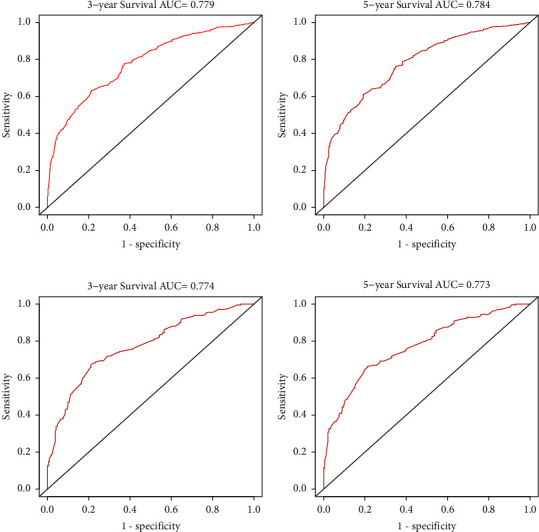
The ROC curves of the nomograms for 3- and 5-year OS prediction of the training cohort (a, b) and internal validation cohort (c, d).

**Figure 6:**
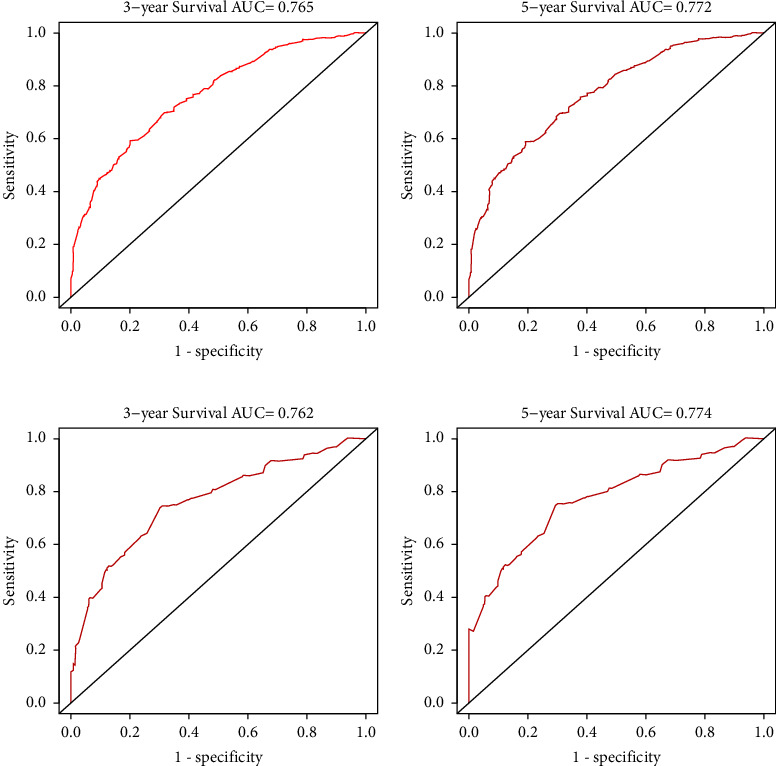
The ROC curves of the nomograms for 3- and 5-year CSS prediction of the training cohort (a, b) and internal validation cohort (c, d).

The C-index of the nomogram for the prediction of OS was 0.764 (95% CI, 0.744–0.784), and CSS was 0.756 (95% CI, 0.732–0.780) in the training cohort (Table 3). In comparison, OS and CSS for the Ann Arbor stage system were just 0.564 and 0.589. The nomogram was then validated in the internal gastric-DLBCL validation cohorts (512 cases). The model also showed a good level of discriminative ability to predict OS (C-index 0.745) and CSS (C-index 0.751). The nomogram was well calibrated, as revealed by the calibration curves (Figures 3 and 4). And it also performed well in predicting OS and CSS of patients with gastric DLBCL (Figures 5 and 6). The AUC of the nomogram for predicting 3- and 5-year OS were 0.779 and 0.784 in the training set, and 0.774 and 0.740 in internal validation set. In terms of 3- and 5-year CSS for the nomogram, the AUC was 0.765 and 0.772 in training set, and 0.762 and 0.774 in internal validation set.

### 3.5. Comparison of the Values

The internal validation cohort calibration curves showed good optimal agreement between prediction by nomogram and observation in the probability of 3- and 5-year survival. As shown in Tables 3 and 4, we further compared the C-index and AUC of the nomogram to the Ann Arbor stage system. The C-index was much lower in Ann Arbor stage system, just 0.562 of OS and 0.552 of CSS in the validation set. In addition, the AUC values of OS and CSS for nomogram also performed much better than the Ann Arbor stage system, particularly the 5-year OS for the training cohort (0.784 of nomogram *versus* 0.578 of Ann Arbor stage system).

## 4. Discussion

Primary gastrointestinal lymphoma (PGIL) is relatively rare and only constitutes less than 5% of gastrointestinal (GI) tract tumors. Primary gastric DLBCL is the most common location of DLBCL in the gastrointestinal tract [8]. Several staging systems have been developed over the past decades to improve prognostic stratification of gastrointestinal lymphoma; unfortunately, there has been no accepted standard till now [9–11]. In this study, we involved the information of 7200 patients diagnosed as PG-DLBCL from the large dataset SEER, and then, collected 1204 cases to construct a novel predictive nomogram and validate it with a 512 patient internal validation cohort. We found the characteristics of patients including age, race, marital status, insurance status, Ann Arbor stage, chemotherapy, and radiation therapy were associated with prognosis. Additionally, this nomogram performed with excellent accuracy as assessed by C-index and AUC. Compared to the Ann Arbor stage scoring system, the C-index of the nomogram for OS and CSS prediction were more accurate both in the training and validation sets, and the AUC values of the nomogram for predicting 3- and 5-year OS and CSS were higher, which can help clinicians accurately predict the survival of individual patients.

In this predictive model, the PG-DLBCL patients who were unmarried or single, insured, and Black showed worse outcomes. Better financial and psychological support may be beneficial for treatments, so married patients were associated with better prognosis. Previous research has described that marital status was independently associated with the 5-year relative survival of patients with DLBCL [12]. According to the majority of previous results, Black patients had worse outcomes, and lower socioeconomic status for Black patients might have contributed to the worse survival [13–15]. Based on these evidences, we concluded the worse survival for Black patients with DLBCL may be associated with religion, habit, and living environment. Although the patients having relatively good financial aid found it easier to follow the treatments, the result was that the PG-DLCBL patients insured showed worse results compared to the patients with insurance (any Medicaid or insured) confused us. We thought there might be a complex interaction among social economics, demographic factors, and cancer outcomes. In general, the results need a large amount of research evidence from the real world.

Since the era of rituximab arrived, the outcomes of DLBCL have been improved. Previous research recommended chemotherapy as the front-line treatment for PGI-DLBCL while surgery was conducted to relieve tumor-related complications or a make diagnosis [16]. Several case reports found that surgical intervention for gastric DLBCL showed a better prognosis [17, 18]. Here, the constructed nomogram confirmed that surgery intervention had no significant association with the prognosis of PG-DLBCL patients. However, the role of radiation therapy in DLBCL was limited in combined modality therapy for DLBCL. Recent evidence demonstrated that selected patients with DLBCL had significantly better outcomes when radiation treatment was added to immunochemotherapy; and Koiwai et al. reported that application of decreased radiation dose might be effective for localized DLBCL patients who showed a good response to chemotherapy [19, 20]. Our research also found radiation therapy might impact the prognosis of patients with PG-DLBCL, but it just played a limited part. Even if chemotherapy was the key to improving the prognosis, treatment regimens were unclear in this retrospective study.

To our knowledge, this is currently the largest retrospective case series of PG-DLBCL with the aim of getting a prognostic model to predict OS and CSS. However, it needs further validation by way of large randomized controlled trials. As the data source, SEER, did not provide the IPI scores of the patients, we cannot compare this nomogram with the IPI scoring system. In addition, the results of this study ignored the genetic characteristics, which are now proved to be important in the diagnosis and prognosis of the disease [21, 22]. Even so, our study remains an instructive and efficient model of PG-DLBCL prognosis.

## 5. Conclusions

We have developed and validated a novel nomogram for predicting OS and CSS in patients with PG-DLBCL, which has never been investigated before. The parameters in the model are routinely evaluated and easily adopted in the clinic, assisting clinicians in making predictions about individual patient survival and providing improved treatment strategies.

## Figures and Tables

**Table 1:** Patient characteristics in the study.

Variables	Total (*n* = 1716)	Training cohort (*n* = 1204)	Validation cohort (*n* = 512)	*P*
*Age (years)*				0.932
<60	517 (30.1)	362 (30.0)	155 (30.3)	
≥60	1199 (69.9)	842 (70.0)	357 (69.7)	

*Sex n (%)*				0.464
Female	710 (41.4)	505 (41.9)	205 (40.0)	
Male	1006 (58.6)	699 (58.1)	307 (60.0)	

*Race n (%)*				0.906
Black	150 (8.7)	106 (8.8)	44 (8.6)	
White	1344 (78.3)	945 (78.5)	399 (77.9)	
Others	222 (13.0)	153 (12.7)	69 (13.5)	

*Marital status n (%)*				0.08
Married	984 (57.3)	674 (56.0)	310 (60.5)	
Unmarried/single	732 (42.7)	530 (44.0)	202 (39.5)	
*Insurance n (%)*				0.586
Insured	1345 (78.4)	947 (78.7)	398 (77.8)	
Any medicaid	295 (17.2)	201 (16.7)	94 (18.3)	
Uninsured	76 (4.4)	56 (4.6)	20 (3.9)	

*Ann Arbor stage n (%)*				0.442
I	719 (41.9)	493 (40.9)	226 (44.1)	
II	390 (22.7)	283 (23.5)	107 (20.9)	
III	146 (8.5)	107 (8.9)	39 (7.6)	
IV	461 (26.9)	321 (26.7)	140 (27.4)	

*Surgery n (%)*				0.925
No	1550 (90.3)	1087 (90.3)	463 (90.4)	
Yes	166 (9.7)	117 (9.7)	49 (9.6)	

*Chemotherapy n (%)*				0.808
No/unknown	419 (24.4)	292 (24.2)	127 (24.8)	
Yes	1297 (75.6)	912 (75.8)	385 (75.2)	

*Radiation n (%)*				0.809
No/unknown	1449 (84.4)	1015 (84.3)	434 (84.8)	
Yes	267 (15.6)	189 (15.7)	78 (15.2)	

*Note.* If *t* ≥ 5, Pearson *X*^2^ test. If 1 ≤ *t* < 5, continuity correction *X*^2^ test; unmarried: include single, divorced, and widowed.

**Table 2:** Univariate and multivariate analysis of survival with PG-DLBCL.

Variables	Overall survival				Cancer-specific survival			
	Univariate analysis		Multivariate analysis		Univariate analysis		Multivariate analysis	
	Log rank *X*^2^	*P*	HR (95% CI)	*P*	Log rank *X*^2^	*P*	HR (95% CI)	*P*
Sex	0.333	0.564			0.012	0.911		
Female								
Male								

Age (years)	223.417	0.001		0.001	117.129	0.001		0.001
<50			Reference				Reference	
50–59			1.076 (0.720–1.607)	0.722			1.027 (0.658–1.603)	0.906
60–69			1.848 (1.284–2.659)	0.001			1.856 (1.245–2.766)	0.002
70–79			2.834 (1.983–4.052)	0.001			2.143 (1.433–3.206)	0.001
≥80			4.223 (2.966–6.013)	0.001			3.225 (2.176–4.779)	0.001

Race	8.717	0.013		0.001	8.260	0.016		0.003
Black			Reference				Reference	
White			0.633 (0.482–0.830)	0.001			0.612 (0.450–0.831)	0.002
Others			0.501 (0.349–0.718)	0.001			0.523 (0.349–0.786)	0.002

Marital status	54.196	0.001			36.632	0.001		
Married			Reference				Reference	
Unmarried/single			1.492 (1.257–1.771)	0.001			1.472 (1.206–1.796)	0.001

Insurance	16.042	0.001		0.006	13.208	0.001		0.017
Insured			Reference				Reference	
Any medicaid			1.362 (1.100–1.686)	0.005			1.345 (1.054–1.716)	0.017
Uninsured			0.724 (0.423–1.240)	0.724			0.681 (0.374–1.241)	0.210

Ann Arbor stage	31.392	0.001		0.001	51.234	0.001		0.001
I			Reference				Reference	
II			1.452 (1.156–1.823)	0.001			1.526 (1.161–2.006)	0.002
III			1.828 (1.354–2.469)	0.001			1.996 (1.400–2.845)	0.001
IV			2.159 (1.758–2.650)	0.001			2.760 (2.176–3.501)	0.001

Surgery	1.967	0.161			0.665	0.415		
No			Reference				Reference	
Yes			1.013 (0.774–1.325)	0.925			0.731 (0.531–1.006)	0.054

Chemotherapy	278.954	0.001			203.315	0.001		
No/unknown			Reference				Reference	
Yes			0.315 (0.260–0.380)	0.001			0.296 (0.239–0.367)	0.001

Radiation	23.686	0.001			19.663	0.001		
No/unknown			Reference				Reference	
Yes			0.665 (0.511–0.865)	0.002			0.656 (0.479–0.900)	0.009

*Note.* Univariate analysis: Kaplan–Meier analysis; multivariate analysis: cox regression analysis; HR: hazard ratio.

**Table 3:** C-index for the nomogram and Ann Arbor stage systems in patients with PG-DLBCL.

Survival		Training cohort	*P*	Internal validation cohort	*P*
*Overall survival*	Nomogram	0.764 (0.744–0.784)	<0.001	0.745 (0.714–0.776)	<0.001
	Ann Arbor stage	0.564 (0.540–0.588)		0.562 (0.513–0.583)	

*Cancer-specific survival*	Nomogram	0.756 (0.732–0.780)	<0.001	0.751 (0.714–0.788)	<0.001
	Ann Arbor stage	0.589 (0.562–0.616)		0.552 (0.509–0.595)	

**Table 4:** The comparison of AUC between nomogram and Ann Arbor stage systems in patients with PG-DLBCL.

Survival		3-year survival AUC (TC)	5-year survival AUC (TC)	3-year survival AUC (IVC)	5-year survival AUC (IVC)
*Overall survival*	Nomogram	0.779	0.784	0.774	0.773
	Ann Arbor stage	0.590	0.578	0.548	0.545

*Cancer-specific survival*	Nomogram	0.765	0.772	0.762	0.774
	Ann Arbor stage	0.612	0.610	0.550	0.541

TC: training cohort; IVC: internal validation cohort.

## Data Availability

Publicly available datasets were analyzed in this study. These data are available in Surveillance, Epidemiology, and End Results (SEER) database (https://seer.cancer.gov/). The datasets generated in this study are available from the corresponding authors upon request.

## Additional Points

All the statistical analyses were performed using R version 3.4.2 software (the R Foundation for Statistical Computing, Vienna, Austria. http://www.r-project.org).
